# Investigating carbon dioxide absorption by urban trees in a new park of Bangkok, Thailand

**DOI:** 10.1186/s12898-020-00289-4

**Published:** 2020-04-13

**Authors:** Pantana Tor-ngern, Nisa Leksungnoen

**Affiliations:** 1 Department of Environmental Science, Faculty of Science, Chulalongkorn University, Bangkok, 10330 Thailand; 2 Environment, Health and Social Data Analytics Research Group, Chulalongkorn University, Bangkok, 10330 Thailand; 3 Department of Forest Biology, Faculty of Forestry, Kasetsart University, Bangkok, 10900 Thailand

**Keywords:** Urban trees, Stomatal conductance, Carbon dioxide absorption

## Abstract

**Background:**

Trees remove atmospheric carbon dioxide through photosynthesis, hereafter CO_2_ absorption (*A*). Despite growing urban green areas, only a few studies have quantified *A* of urban trees and assessed their dynamical changes with varying atmospheric conditions. Hence, we investigated *A* in nine dominant tree species in a new park of Bangkok.

**Results:**

Results revealed that *A* of two tree species (*Millingtonia hortensis* and *Afzelia xylocarpa*) significantly increased with vapor pressure deficit (VPD) until it reached a maximum and declined when VPD decreased, with no seasonal difference. Five of them (*Dalbergia cochinchinensis, Tabebuia rosea, Lagerstroemia floribunda, Dipterocarpus alatus* and *Bauhinia purpurea*) exhibited different response patterns of *A* to VPD between wet and dry seasons. In contrast, the *A* of two tree species (*Samanea saman* and *Homalium tomentosum*) did not respond to changing VPD in either season.

**Conclusions:**

Comparing planting scenarios of insensitive (i.e. no response to VPD) versus sensitive (i.e. significant response to VPD) species, we found that planting a mixture of sensitive and insensitive tree species would improve the park’s capacity of *A* across seasons, allowing climate change adaptation to adverse environmental impacts such as droughts and the urban heat island effects, and would increase biodiversity. Additionally, planting insensitive tree species would significantly increase the capacity of the park for CO_2_ mitigation. These findings are useful for those who design parks and expand urban green areas to fully benefit ecosystem services from trees.

## Background

Cities experience many adverse environmental impacts including intensified warming, due to the heat island effect [1] and high atmospheric carbon dioxide (CO_2_) which prevents heat emission from the earth surface. Consequently, urban greening has been applied to mitigate the rising atmospheric CO_2_ [2–5]. Urban greening includes planting trees along the streets, park creation, and other ways that increase the green space in cities, which also provide other ecosystem services, such as clean air, shade and cooling effects, recreational and educational values [6, 7]. Trees make up the bulk of biomass of green space in cities and may be an effective choice to acquire significant CO_2_ mitigation, namely CO_2_ absorption (*A*) through photosynthesis. However, different tree species, and of different ages, respond to the environments differently [8, 9] and therefore may absorb CO_2_ at different rates. Hence, investigating the species-specific responses of urban trees to environmental conditions will improve our understanding of how different urban tree species provide the ecosystem service of CO_2_ mitigation through photosynthesis, which is needed for effective planning and management of green space to optimize land-use in the urban areas.

With these regards, we investigated the responses of *A* by urban trees through stomatal changes with varying atmospheric conditions. Specifically, we measured stomatal conductance (*g*_*s*_), which is a variable showing stomatal responses to changing environments, on nine dominant tree species at a newly established park in Bangkok in wet and dry seasons and estimated *A* using *g*_*s*_ based on a diffusion equation. Then, we characterized the tree species-specific responses of *A* to changing atmospheric conditions. Results provide useful information for selective planting of urban trees to optimize CO_2_ mitigation services of green space in cities.

## Results

**Fig. 1:**
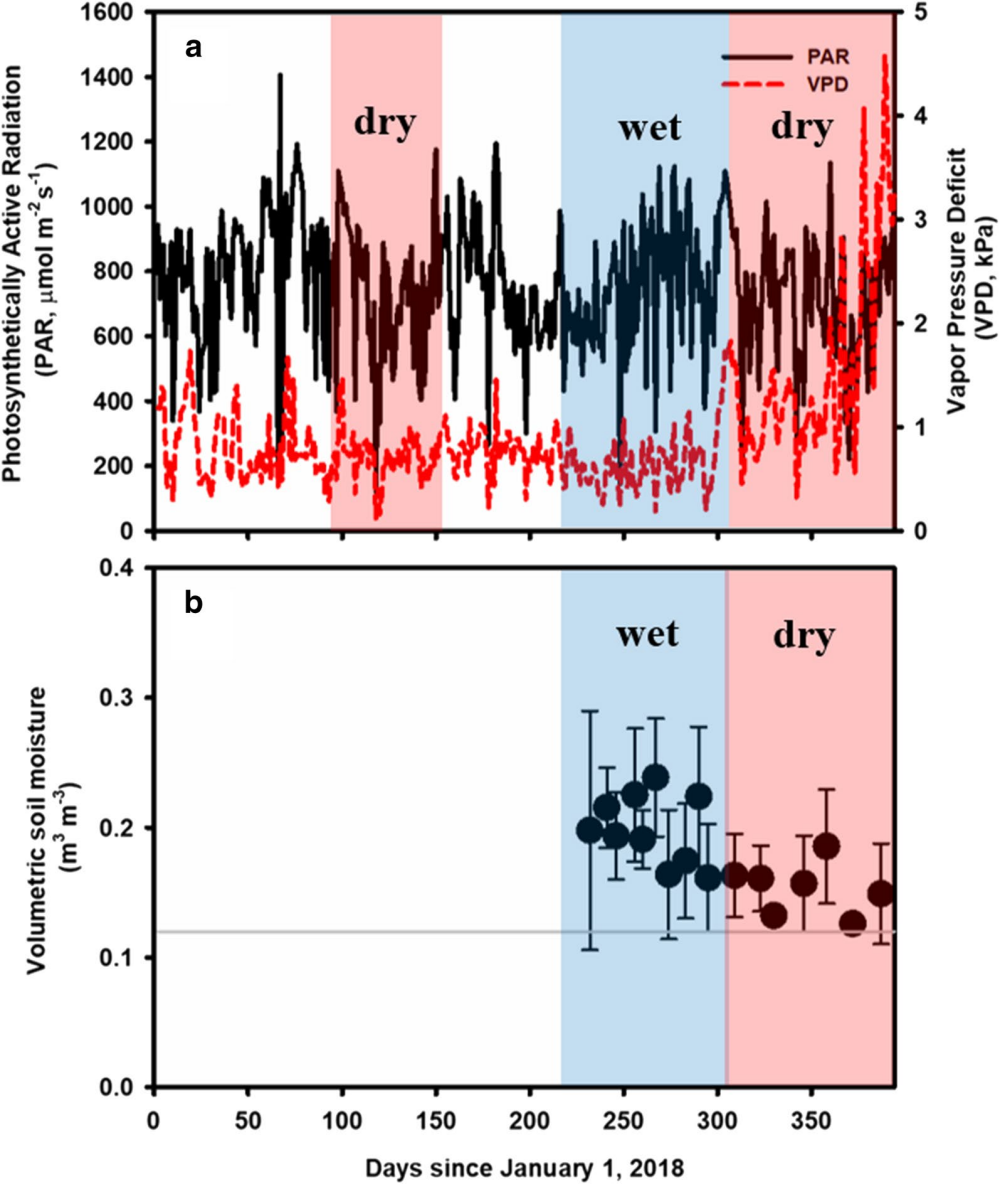
Environmental conditions for the study site, covering the periods of data collection. **a** Daily vapor pressure deficit (VPD) in kPa is shown as a red dashed line and sunlight, represented by photosynthetically active radiation (PAR), is displayed as a black solid line. **b** Average values of volumetric soil moisture (m^3^ m^−3^) are displayed as solid points with one standard deviation shown as an error bar. Gray line represents 70% of the field capacity of the soil at our site. Blue and red shaded regions show collection period in the wet and dry seasons, respectively

Figure 1 shows environmental conditions at our site, including vapor pressure deficit (VPD), sunlight expressed as photosynthetically active radiation (PAR) and soil moisture. The PAR did not vary across the collection periods in both seasons (t-test, p = 0.34, Fig. 1a) and was relatively high, ranging 850–1000 µmol m^−2^ s^−1^ during the measurement days. The VPD was the only environmental variable that display seasonal variation (t-test, p < 0.0001). Volumetric soil moisture was significantly higher than 70% of the field capacity (one-sample t-test, p = 0.002) and was not different between wet and dry seasons (t-test, p = 0.19, Fig. 1b).

**Fig. 2:**
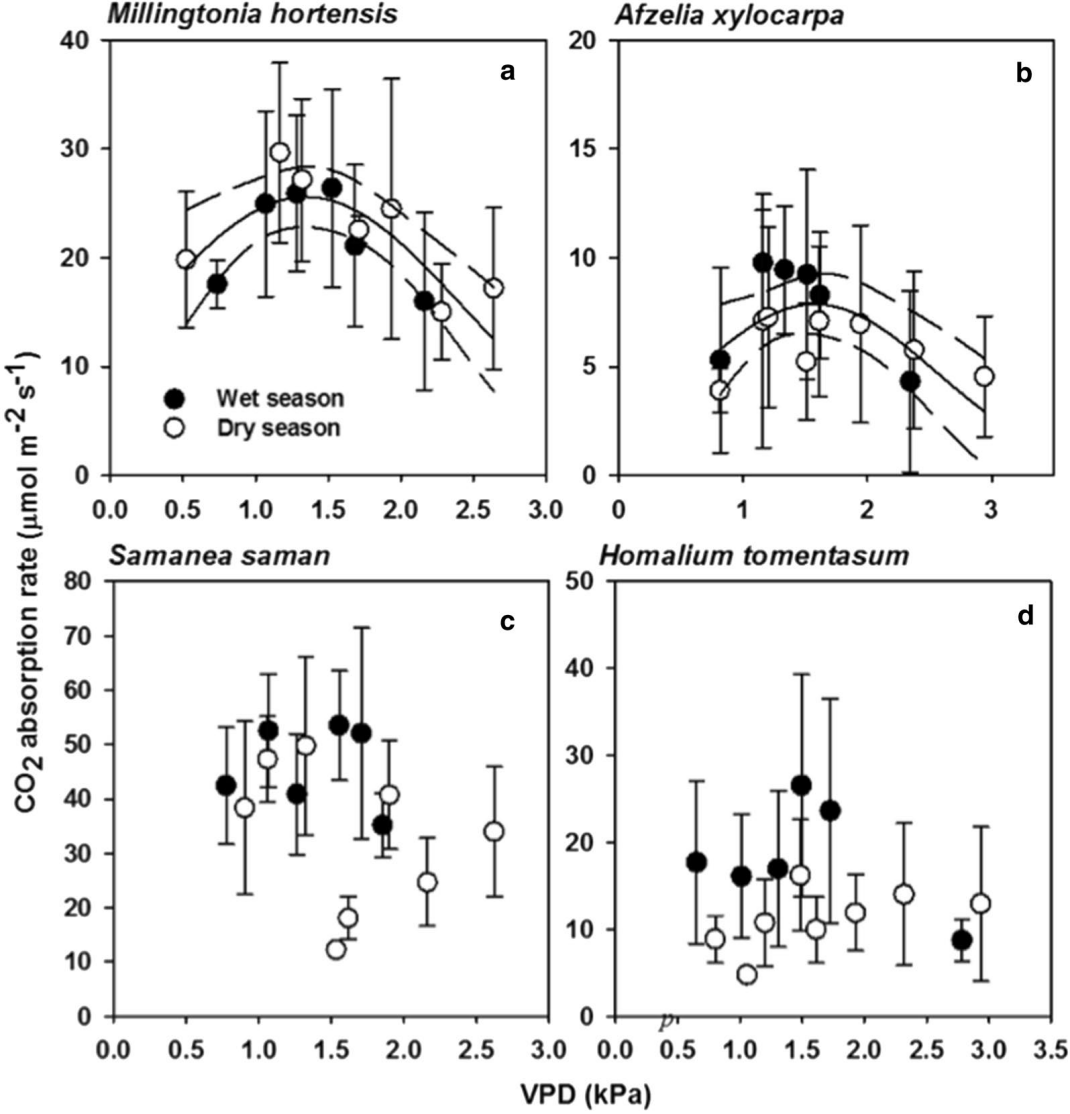
Tree species with sensitive responses of the CO_2_ absorption (*A*, μmol m^−2^ s^−1^) to vapor pressure deficit (VPD, kPa) in both wet and dry seasons (**a**, **b**) and those with insensitive responses (**c**, **d**). Closed (open) symbols represent *A* averages for the wet (dry) season with one standard deviation shown as error bars. Solid lines show significant regression patterns at 0.05 level with 95% confidence intervals shown as dashed lines. Regression equations are listed in Table 1

Regression analyses showed different results among these species. The CO_2_ absorption of *Millingtonia hortensis* and *Afzelia xylocarpa* displayed quadratic changes with VPD, increasing at low VPD values until about 1.3–1.6 kPa and decreasing afterwards with no seasonal difference (Fig. 2a, b; regression equations are presented in Table 1). Among the studied tree species, only *Samanea saman* and *Homalium tomentosum* showed no significant responses of *A* to VPD in either season (Fig. 2c, d; p ≥ 0.19).

**Table 1:** Regression equations of the CO_2_ absorption responses to vapor pressure deficit

Species	Season	Fitting equation	*r*^*2*^	*p*	*n*
*Millingtonia hortensis*	Both	$y = 25.59 \times \exp \left( { - 0.5 \times \left( {\frac{x - 1.35}{1.08}} \right)^{2} } \right)$	0.63	0.007	13
*Afzelia xylocarpa*	Both	$y = 7.87 \times \exp \left( { - 0.5 \times \left( {\frac{x - 1.58}{0.97}} \right)^{2} } \right)$	0.43	0.04	14
*Dalbergia cochinchinensis*	Dry	$y = 10.22 - 4.3 \times \ln (x)$	0.65	0.016	8
*Tabebuia rosea*	Dry	$y = 33.61 - 14.79 \times \ln (x)$	0.86	0.003	7
*Lagerstroemia floribunda*	Wet	$y = - 4.61 + 36.95x - 8.31x^{2}$	0.61	0.02	7
*Dipterocarpus alatus*	Wet	$y = 24.7 \times \exp \left( { - 0.5 \times \left( {\frac{x - 1.2}{0.69}} \right)^{2} } \right)$	0.81	0.03	6
*Bauhinia purpurea*	Wet	$y = 25.2 \times \exp \left( { - 0.5 \times \left( {\frac{x - 1.71}{0.81}} \right)^{2} } \right)$	0.84	0.03	6

**Fig. 3:**
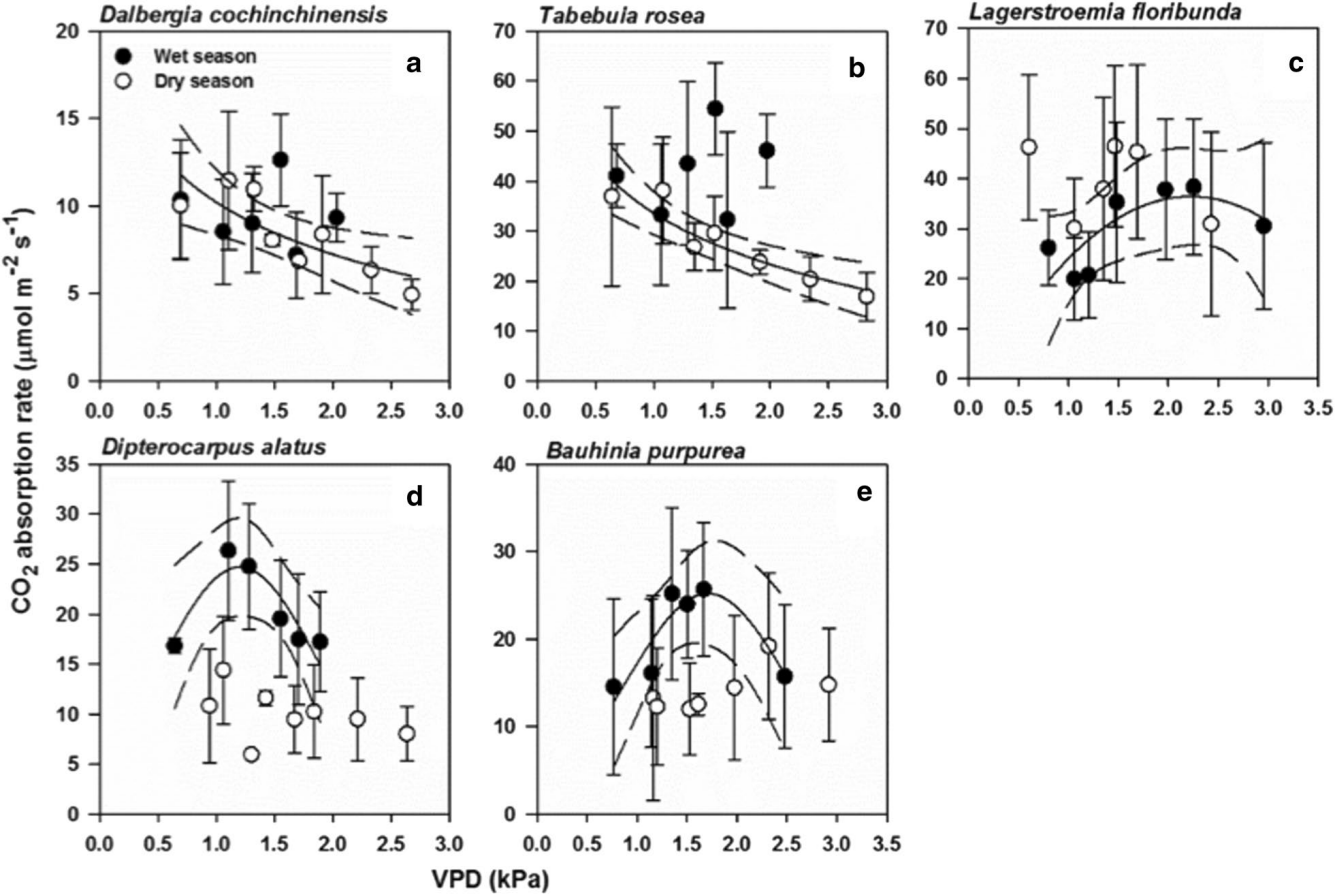
Tree species with seasonally sensitive responses of the CO_2_ absorption (*A*, μmol m^−2^ s^−1^) to vapor pressure deficit (VPD, kPa). Species with sensitive responses in the dry season only (**a**, **b**) and those with sensitive responses in the wet season only (**c**–**e**). Closed (open) symbols represent *A* averages for the wet (dry) season with one standard deviation shown as error bars. Solid lines show significant regression at 0.05 level with 95% confidence intervals shown as dashed lines. Regression equations are listed in Table 1

In the dry season, *Dalbergia cochinchinensis* and *Tabebuia rosea* decreased logarithmically with increasing VPD (Fig. 3a, b; open symbols, Table 1). However, *A* of the two tree species did not change with VPD in the wet season (Fig. 3a, b; closed symbols, p ≥ 0.61). In contrast, *A* of *Lagerstroemia floribunda, Dipterocarpus alatus* and *Bauhinia purpurea* responded to VPD in the wet season only. In *Lagerstroemia floribunda*, *A* increased at low VPD, reaching a maximum value at VPD of 2.1 kPa and declined afterwards (Fig. 3c; closed symbols, Table 1) while no response of *A* was observed in the dry season (Fig. 3c; open symbols, p = 0.76). *Dipterocarpus alatus* and *Bauhinia purpurea* in the wet season significantly changed with increasing VPD in quadratic patterns, with a maximum *A* at VPD of 1.1–1.6 kPa (Fig. 3d, e; closed symbols, Table 2) whereas no significant responses to VPD were observed in either species in the dry season (Fig. 3d, e; open symbols, p ≥ 0.1).

**Table 2:** Characteristics of trees that were selected for measurements

Species	DBH (cm)	H (m)	LAI (wet season)	LAI (dry season)
*Millingtonia hortensis*	13.5 ± 0.7	6.7 ± 0.4	1.03 ± 0.30	0.86 ± 0.20
*Afzelia xylocarpa*	14.2 ± 2.8	6.2 ± 0.5	1.12 ± 0.21	0.61 ± 0.13
*Dalbergia cochinchinensis*	10.8 ± 1.3	6.5 ± 0.5	1.58 ± 0.24	1.33 ± 0.41
*Tabebuia rosea*	10.1 ± 1.8	5.4 ± 0.6	0.58 ± 0.24	0.47 ± 0.20
*Lagerstroemia floribunda*	11.7 ± 1.1	6.0 ± 0.4	0.79 ± 0.43	0.80 ± 0.31
*Dipterocarpus alatus*	12.5 ± 2.2	6.0 ± 0.7	0.92 ± 0.35	0.31 ± 0.11
*Bauhinia purpurea*	9.7 ± 2.8	6.8 ± 3.1	1.55 ± 0.42	1.36 ± 0.49
*Homalium tomentosum*	10.6 ± 2.3	7.7 ± 0.4	0.76 ± 0.39	0.65 ± 0.13
*Samanea saman*	13.7 ± 1.4	5.7 ± 0.4	1.50 ± 0.61	1.41 ± 0.30

Values are averages and one standard deviation

DBH is diameter at breast height in cm, H is tree height in m and LAI is leaf area index

## Discussion

The assumption that soil water availability was sufficient for plant growth during the study period was verified. Consequently, we considered VPD as the only driver of stomatal changes, and hence *A*, in our analyses. Overall, *A* responded to VPD differently among the nine tree species. Carbon dioxide absorption (*A*) was higher in the wet season than in the dry season in all tree species, which may be attributed to decreased stomatal opening under high VPD conditions in the dry season as normally observed in several urban tree species [10]. The *A* responses of the nine tree species can be categorized into those that were sensitive and insensitive to VPD as follows.

The significant responses of *A* in *Millingtonia hortensis* and *Afzelia xylocarpa* to VPD, when analyzed with pooled data, suggests that both tree species were highly sensitive to varying atmospheric humidity regardless of the seasons. In contrast, the insignificant responses of A to VPD in *Samanea saman* and *Homalium tomentosum* may be advantageous because both tree species can absorb CO_2_ throughout the year, regardless of the changing atmospheric humidity. However, the magnitude of *A* in *Samanea saman* was higher than that of *Homalium tomentosum*, implying that *Samanea saman* may be suitable for maximizing CO_2_ mitigation in the park.

Some tree species showed seasonal difference in the *A* response to VPD. For *Dalbergia cochinchinensis* and *Tabebuia rosea, A,* in the dry season, logarithmically decreased with VPD, which is typical for plants whose stomatal closure occurs when VPD rises to prevent water loss [11]. However, both species did not respond to VPD in the wet season, implying that *A* was constant regardless of atmospheric conditions. In contrast, *A* of *Lagerstroemia floribunda, Dipterocarpus alatus* and *Bauhinia purpurea* only responded to VPD in the wet season and no patterns were observed in the dry season.

**Fig. 4:**
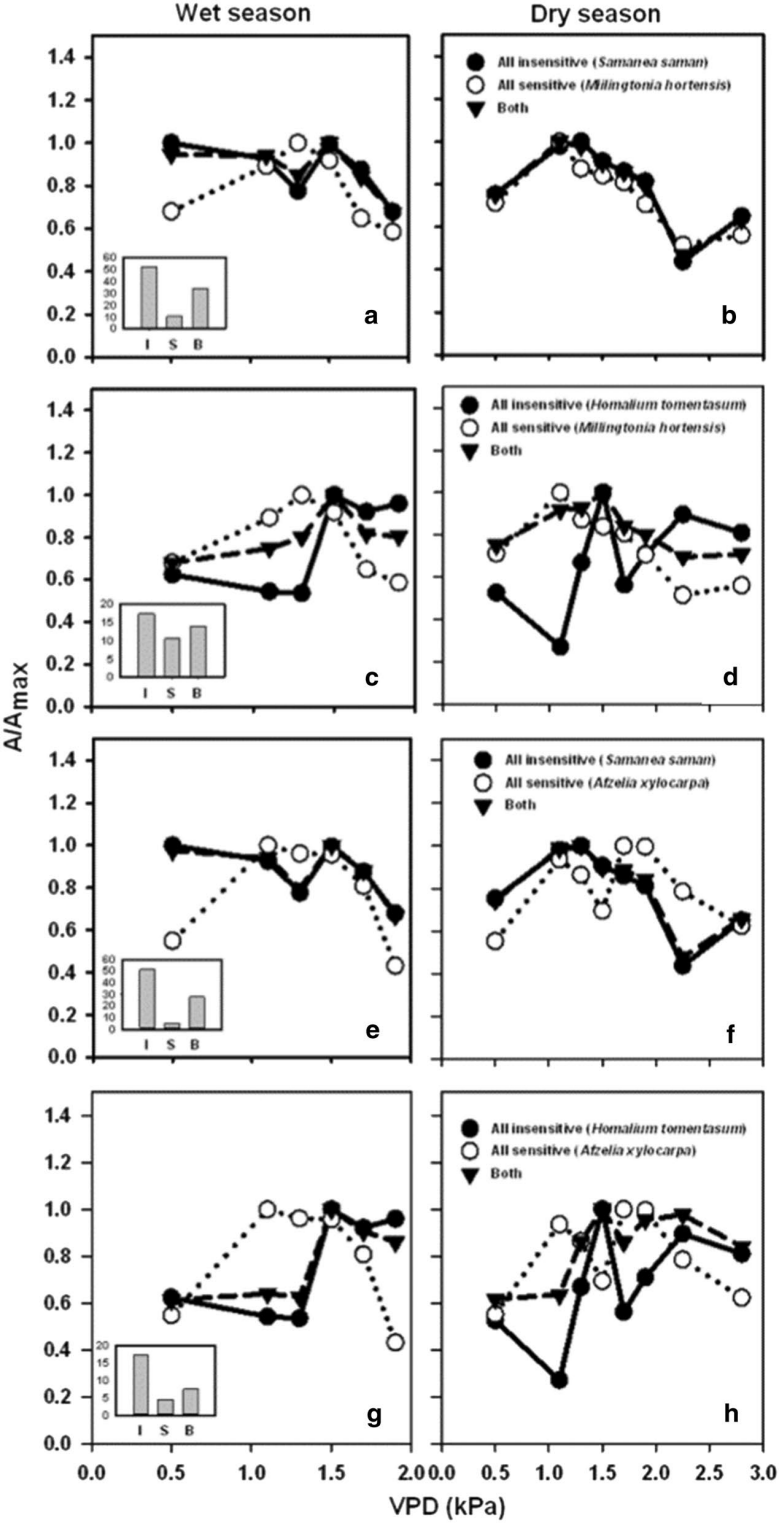
CO_2_ absorption rate relative to the maximum (A/A_max_) by the entire park across VPD ranges with all insensitive species (I, closed circles), all sensitive species (S, open circles) and half of each of insensitive and sensitive species (B, lower triangles) in the wet (**a**, **c**, **e**, **g**) and dry (**b**, **d**, **f**, **h**) seasons. Insets (small bar graphs) show the total CO_2_ absorption (500 trees) in t CO_2_ per year in each corresponding scenario

To illustrate the application of these results for selective planting, we performed hypothetical analyses by analyzing *A* of the entire park, assuming 500 trees were planted, under three scenarios (1) the park consists of one insensitive tree species only (insensitive; I) (2) the park contains one sensitive tree species only (sensitive; S) and (3) half of the park is occupied by one sensitive and one insensitive tree species (both; B). Based on our results, the insensitive tree species are *Samanea saman* and *Homalium tomentosum* whereas the sensitive tree species are *Millingtonia hortensis* and *Afzelia xylocarpa*. In each scenario, we considered the variations of relative *A* to the maximum (A/A_max_) with VPD in the wet and the dry season (Fig. 4) because magnitudes of A in the compared tree species were different. Additionally, we determined the absolute values of *A* which represent the amount of CO_2_ absorbed by trees in each hypothetical park (insets in Fig. 4).

**Fig. 5:**
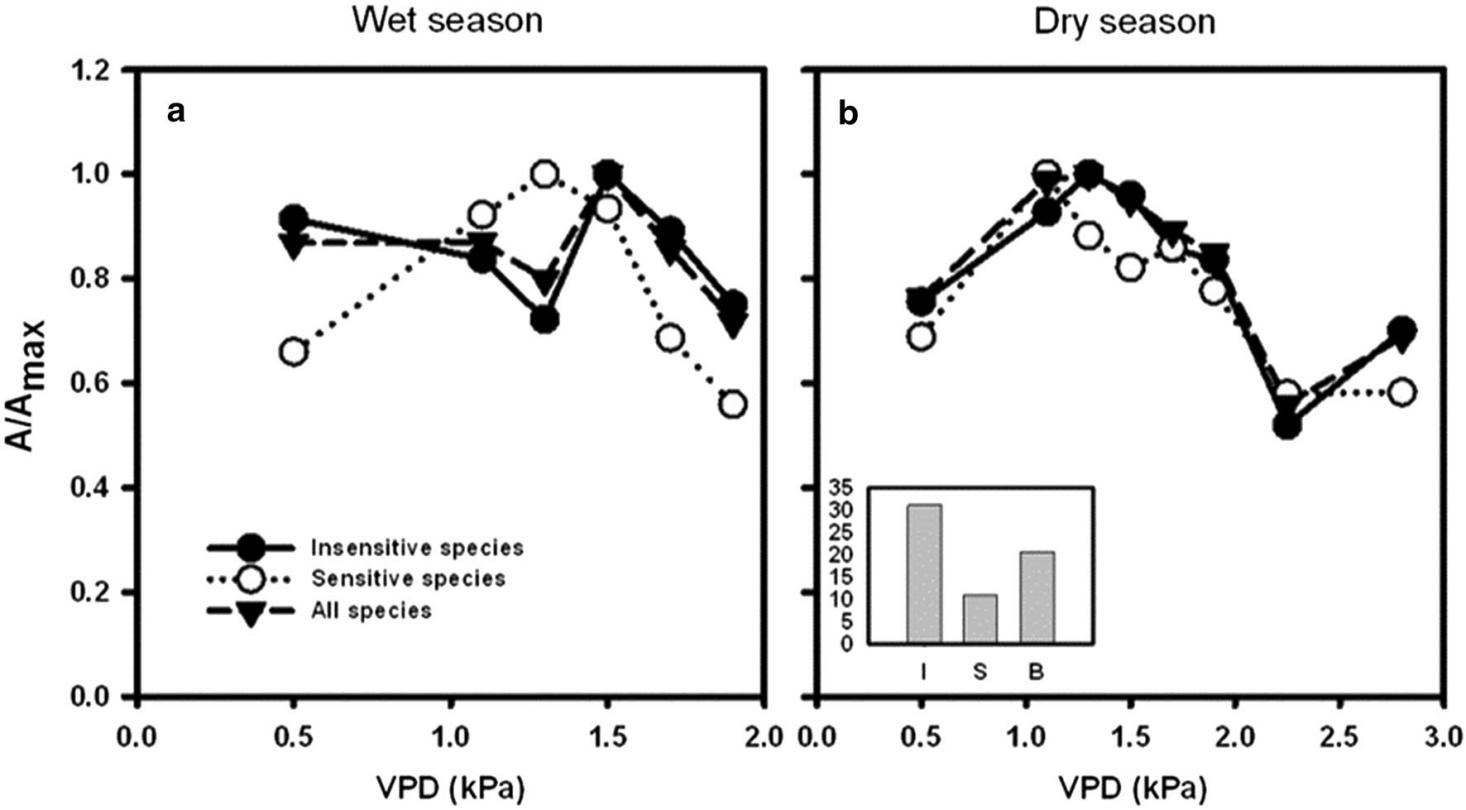
CO_2_ absorption rate relative to the maximum (A/A_max_) by the entire park across VPD ranges with all insensitive tree species (I, closed circles), all sensitive tree species (S, open circles) and half of each of insensitive and sensitive tree species (B, lower triangles) in the wet (**a**) and dry (**b**) seasons. Insets (the small bar graph) show the total CO_2_ absorption (500 trees) in t CO_2_ per year in each corresponding scenario. In this case, I includes *Samanea saman* and *Homalium tomentosum* whereas S includes *Millingtonia hortensis* and *Afzelia xylocarpa* species

Overall, variations of A/A_max_ in *B* followed those in *I* (Fig. 4; compare closed circles and lower triangles), especially when *I* was *Samanea saman* (Fig. 4b, f) because this species yielded the highest *A*. In the case of when insensitive tree species was *Homalium tomentosum*, the patterns of A/A_max_ in *B* were also similar to *I* but the magnitudes increased compared to *I* (Fig. 4c, d, g, h). Similarly, in the wet season, overall variations of A/A_max_ in B remained relatively closer to one compared to S scenario, except in the scenario that involves *Homalium tomentosum* and *Afzelia xylocarpa* (Fig. 4g). These results suggest that *B* improved the capacity of CO_2_ absorption by the park across seasons since A/A_max_ is maintained relatively close to one across the VPD range. The amount of CO_2_ absorption by the park is ranked, from the highest to the lowest, as I > B > S (insets in Fig. 4). This means that planting only insensitive tree species in the park would result in a maximum total CO_2_ absorption but the capacity for the absorption under changing atmospheric conditions, such as VPD, would be optimized with mixed insensitive and sensitive tree species. We further explored this idea by including all four tree species in the analyses. In this case, we combined *Samanea saman* and *Homalium tomentosum* as I, *Millingtonia hortensis* and *Afzelia xylocarpa* as S and all four tree species as B. The analysis showed the same result with A/A_max_ variations B following I with improved magnitudes (Fig. 5). Thus, our hypothetical analyses revealed that planting insensitive tree species would maximize CO_2_ absorption and therefore enhancing the role of CO_2_ mitigation by parks. With the mixture of sensitive and insensitive tree species, the park would maintain relatively high CO_2_ absorption rates compared to the maximum, especially in the dry season when the atmospheric humidity is usually low. This selective planting scheme is suitable for designing parks that would tolerate adverse climate change impacts, such as greater frequency and intensity of droughts and the intensified warming in cities. Planting mixed tree species would also increase biodiversity in the park which may induce other useful ecosystem services, such as habitats for various animal species and beautiful scenic views due to different leaf shapes, crowns and flowers. Nevertheless, further detailed studies on the physiological responses of these species to atmospheric conditions should be performed to confirm such findings.

## Conclusions

This study investigated specific responses of CO_2_ absorption by nine urban tree species in a newly established park in Bangkok, Thailand. Results revealed that CO_2_ absorption of two tree species (*Millingtonia hortensis* and *Afzelia xylocarpa*) significantly changed with vapor pressure deficit with no seasonal difference. Five of them (*Dalbergia cochinchinensis, Tabebuia rosea, Lagerstroemia floribunda, Dipterocarpus alatus* and *Bauhinia purpurea*) exhibited different responses of CO_2_ absorption to vapor pressure deficit between wet and dry seasons. In contrast, the CO_2_ absorption of two tree species (*Samanea saman* and *Homalium tomentosum*) did not respond to changing vapor pressure deficit in each season. These results are of great value because data of CO_2_ absorption and its responses to atmospheric conditions by urban trees in the tropics are still rare. The hypothetical analyses suggested that planting a mixture of sensitive and insensitive tree species would improve the park’s capacity of CO_2_ absorption across seasons, allowing climate change adaptation to adverse environmental impacts including droughts and the urban heat island effects, and would increase biodiversity. Additionally, planting insensitive tree species would maximize the capacity of the park for CO_2_ mitigation. Nevertheless, it should be noted that this result is based on simple measurements and calculations and muse not be applied in practical situations until further detailed investigations on plant physiology are performed.

## Methods

### Study area

The study was conducted in the Chulalongkorn University Centenary Park (CU100), a newly established park in Bangkok (13° 44′ 02.9″N 100° 31′ 54.1″E). The elevation is 1.5 m asl [12]. According to a 30-year record of climatological data (1981–2010) at a Bangkok metropolis station (Thai Meteorological Department), the mean annual air temperature is 28.6 °C with extreme maximum temperature in summer up to 39.7 °C and extreme minimum temperature in winter down to 12 °C and the mean annual rainfall is 1648 mm. The 4.48-ha park was established in 2016 and includes various types of vegetation. Based on a tree survey in February 2017, there are 706 trees of 48 species in this park. The diameter at breast height and tree height are, on average, 11.16 ± 3.39 cm and 6.73 ± 1.55 m, respectively (Table 2).

### Environmental variables

Stomatal conductance is regulated by environmental factors, including air humidity, temperature, sunlight, and soil moisture. We obtained air temperature and relative humidity data from a nearby station (Air Quality and Noise Management Division of the Pollution Control Department, Thailand). The two variables were used to determine vapor pressure deficit (VPD, kPa) which indicates atmospheric humidity such that dry (wet) air corresponds to high (low) VPD.

Vapor pressure deficit is the difference between saturated vapor pressure and actual vapor pressure in the air, a variable called vapor pressure deficit (VPD, kPa), and is calculated by

1 $$ {\text{VPD = }}\left( { 1 - \frac{\text{RH}}{ 1 0 0}} \right) \times {\text{SVP }} $$

where RH is relative humidity (%) and SVP (kPa) is the saturated vapor pressure which is expressed as

2 $$ {\text{SVP}} = 6 1 0. 7 \times 1 0  ^{{\frac{{ 7. 5 {\text{T}}}}{{ 2 3 7. 5 {\text{ + T}}}}}} $$

where T represents air temperature in °C [13].

Sunlight was not measured at the meteorological station, but we assumed that it was not limiting because measurements were made during daytime on sunny days. To verify this assumption, we referred to photosynthetically active radiation (PAR) from another station within 5 km distance from the site. Because the park is maintained by frequent irrigation, we assumed that soil water availability was at sufficient level for plant growth (more than 70% of the field capacity of the soil) during the study period. To confirm this assumption, volumetric soil moisture was measured by collecting soil samples and measuring water content once a week to validate the assumption. Five soil samples at 5 cm depth were randomly collected at the park using soil core with 15 cm diameter and 15 cm length. Then, the soil samples were weighed for wet mass ($m_{soil.wet}$, kg) and dried at 105 °C for 24 h, or until the weight was constant, for dry mass ($m_{soil.dry}$, kg). Bulk density ($\rho ;$ kg m^−3^) was also estimated as the fraction of dry mass and volume of the soil core. Then, volumetric soil moisture ($\theta_{v}$, m^3^ m^−3^) was computed as

3 $$ \theta_{v} = \frac{{\theta_{m} \times \rho }}{{\rho_{w} }} $$

where

4 $$ \theta_{m} = \frac{{m_{soil.wet} - m_{soil.dry} }}{{m_{soil.dry} }} $$

and $\rho_{w}$ is density of water which is equal to 1000 kg m^−3^ [14]. The field capacity ($\theta_{FC}$) was determined by randomly collecting five soil samples from the park using the same soil core and soaking the soils for 24 h. Then, water was drained from the soils by gravitation and the soils were weighed for wet mass. After that, the soils were oven-dried at 105 °C for 24 h or until the weight was constant, for dry mass. Finally, $\theta_{FC}$ was estimated using the same approach as $\theta_{v}$.

### Stomatal conductance (g_s_) and CO_2_ absorption (A)

Because stomata regulate gas exchanges between plants and the atmosphere [15], we measured leaf stomatal conductance (*g*_*s*_, mmol m^−2^ s^−1^), which is a variable representing stomatal opening in response to weather conditions, using a leaf porometer (SC-1, METER Group, Inc., Pullman, WA, USA). Nine dominant tree species were selected based on ranking of basal areas for this measurement. The tree species include *Millingtonia hortensis, Afzelia xylocarpa, Samanea saman, Homalium tomentosum, Dalbergia cochinchinensis, Tabebuia rosea, Lagerstroemia floribunda, Dipterocarpus alatus* and *Bauhinia purpurea.* For each tree species, five individuals were chosen and three fully-expanded sun leaves, which means leaves that were fully exposed to sunlight, were randomly selected from each individual. We chose leaves in the bottom branch and away from the stem to ensure no shading by adjacent leaves were possible. The measurements were performed every 2-h interval from 7:00 to 17:00, three times in the wet (August–October 2018) and dry (April–May 2018 and November 2018–January 2019) season. Then, *A* (µmol m^−2^ s^−1^) at tree level was calculated as [16]

5 $$ A = 0.001 \times g_{c} \times C_{a} \times \left( {1 - \frac{{C_{i} }}{{C_{a} }}} \right) \times LAI $$

where *g*_*c*_ (mmol m^−2^ s^−1^) is stomatal conductance to CO_2_ and is equal to *g*_*s*_/1.6, *C*_*a*_ is atmospheric CO_2_ concentration (µmol mol^−1^). The *C*_*a*_ value was assumed to be equal to 400 µmol mol^−1^ and this was within the range of atmospheric CO_2_ (395–412 µmol mol^−1^) as monitored in another station within 5 km from the site during the study period. *C*_*i*_/*C*_*a*_ is the ratio between leaf intercellular and atmospheric CO_2_ concentration which is species-specific and was measured using a portable photosynthesis system (TARGAS-1, PP Systems, Amesbury, MA, USA). The ratio was determined using readings of *C*_*i*_ and *C*_*a*_ from the TARGAS-1 system based on the following principle. The intercellular CO_2_ concentration (*C*_*i*_) is calculated using the equation [17]

6 $$ C_{i} \left( {\upmu{\text{mol}}\;{\text{mol}}^{ - 1} } \right) = \frac{{\left[ {\left( {g_{c} - \frac{E}{2}} \right) \times C_{out} } \right] - A_{net} }}{{\left( {g_{c} + \frac{E}{2}} \right)}} $$

where *C*_*out*_ is CO_2_ concentration of the air leaving the cuvette, *E* is transpiration rate calculated from the partial pressures of water vapor of the air entering and exiting the cuvette, *A*_*net*_ is net photosynthesis calculated from the difference between CO_2_ concentrations entering and exiting the cuvette, and *g*_*c*_ is the total conductance to CO_2_ transfer and is expressed as

7 $$ g_{c} \left( {{\text{mmol}}\;{\text{m}}^{ - 2} \;{\text{s}}^{ - 1} } \right) = \left[ {\frac{1}{{\left( {1.585 \times r_{s} } \right) + \left( {1.37 \times r_{b} } \right)}}} \right] \times 10^{3} $$

where *r*_*s*_ is the stomatal resistance of the leave, *r*_*b*_ is the boundary layer resistance, 1.585 represents the diffusion ratio of CO_2_ and water in air and 1.37 is the diffusion ratio of CO_2_ and water in the boundary layer. Leaf area index (LAI) is the leaf area per unit ground area and was obtained in each season using a plant canopy analyzer (LAI-2200C, LI-COR, Lincoln, NE, USA). Note that, because measurements were performed on sunlit leaves (although it appeared that most leaves were sunlit in these trees), the whole-tree *A* refers to the maximum CO_2_ absorption of each tree.

### Data analyses

We assessed *A* responses to VPD using regression of various models based on the patterns of data we observed from exploratory data analysis, separately for each season. Then, we performed an F-test to compare the regression patterns between both seasons. Mean comparisons of environmental data between seasons were assessed using t-test. Calculations and analyses were performed in MATLAB 2017b, The MathWorks, Inc., Natick, MA USA and SigmaPlot version 12.0 from Systat Software, Inc., San Jose, CA USA. Statistical comparison was performed in IBM SPSS Statistics for Windows, Version 22.0. Armonk, NY USA.

## Data Availability

The datasets used and/or analyzed during the current study are available from the corresponding author on reasonable request.

## Abbreviations

*A*: CO_2_ absorption rate
A_max_: Maximum CO_2_ absorption rate
*g*_*s*_: Stomatal conductance
VDP: Vapor pressure deficit
RH: Relative humidity
PAR: Photosynthetically active radiation
SVP: Saturated vapor pressure
T: Air temperature
g_c_: Stomatal conductance to CO_2_
C_a_: Ambient CO_2_ concentration
C_i_: Leaf-intercellular CO_2_ concentration
LAI: Leaf area index

## References

[1] Li D, Bou-Zeid E (2013). Synergistic interactions between urban heat islands and heat waves: the impact in cities is larger than the sum of its parts. J Appl Meteorol Clim.

[2] Escobedo FJ, Kroeger T, Wagner JE (2011). Urban forests and pollution mitigation: analyzing ecosystem services and disservices. Environ Pollut.

[3] Konarska J, Uddling J, Holmer B, Lutz M, Lindberg F, Pleijel H (2016). Transpiration of urban trees and its cooling effect in a high latitude city. Int J Biometeorol.

[4] Selmi W, Weber C, Rivière E, Blond N, Mehdi L, Nowak DJ (2016). Air pollution removal by trees in public green spaces in Strasbourg city, France. Urban For Urban Green.

[5] Velasco E, Roth M, Norford L, Molina LT (2016). Does urban vegetation enhance carbon sequestration?. Landsc Urban Plan.

[6] Akbari H (2002). Shade trees reduce building energy use and CO_2_ emissions from power plants. Environ Pollut.

[7] Pataki DE, Alig RJ, Fung AS, Golubiewski NE, Kennedy CA, McPherson EG (2006). Urban ecosystems and the North American carbon cycle. Glob Change Biol.

[8] Bond BJ (2000). Age-related changes in photosynthesis of woody plants. Trends Plant Sci.

[9] Dierick D, Hölscher D (2009). Species-specific tree water use characteristics in reforestation stands in the Philippines. Agric For Meteorol.

[10] Gillner S, Korn S, Roloff A (2015). Leaf-gas exchange of five tree species at urban street sites. Arboric Urban For.

[11] Oren R, Sperry JS, Katul GG, Pataki DE, Ewers BE, Phillips N (1999). Survey and synthesis of intra- and interspecific variation in stomatal sensitivity to vapour pressure deficit. Plant Cell Environ.

[12] Sinsakul S (2000). Late quaternary geology of the lower central plain, Thailand. J Asian Earth Sci.

[13] Monteith JL, Unsworth MH (1990). Principles of environmental physics.

[14] Brady NC, Well RR (2008). The nature and properties of soils.

[15] Farquhar GD, Sharkey TD (1982). Stomatal conductance and photosynthesis. Ann Rev Plant Physiol.

[16] Katul GG, Ellsworth DS, Lai C-T (2000). Modelling assimilation and intercellular CO_2_ from measured conductance: a synthesis of approaches. Plant Cell Environ.

[17] Von Caemmerer S, Farquhar GD (1981). Some relationships between the biochemistry of photosynthesis and the gas exchange of leaves. Planta.

